# Progress in UV Photodetectors Based on ZnO Nanomaterials: A Review of the Detection Mechanisms and Their Improvement

**DOI:** 10.3390/nano15090644

**Published:** 2025-04-24

**Authors:** Gaoda Li, Bolang Cheng, Haibo Zhang, Xinghua Zhu, Dingyu Yang

**Affiliations:** 1 College of Optoelectronic Technology, Chengdu University of Information Technology, Chengdu 610225, China; ligaoda@cuit.edu.cn (G.L.); zhb@cuit.edu.cn (H.Z.); 2 School of Physics and Optoelectronics, Xiangtan University, Xiangtan 411105, China; bolangcheng@xtu.edu.cn; 3 Dazhou Industrial Technology Research Institute, Dazhou 635000, China; 4 School of Materials Science and Engineering, Xihua University, Chengdu 610039, China; 5 Intelligent Manufacturing Industry Technology Research Institute, Sichuan University of Arts and Science, Dazhou 635000, China

**Keywords:** UV photodetectors, ZnO, photoconductivity, surface depletion, surface barrier

## Abstract

Recent advancements in ultraviolet (UV) photodetection technology have driven intensive research on zinc oxide (ZnO) nanomaterials due to their exceptional optoelectronic properties. This review systematically examines the fundamental detection mechanisms in ZnO-based UV photodetectors (UVPDs), including photoconductivity effects, the threshold dimension phenomenon and light-modulated interface barriers. Based on these mechanisms, a large surface barrier due to surface-adsorbed O_2_ is generally constructed to achieve a high sensitivity. However, this improvement is obtained with a decrease in response speed due to the slow desorption and re-adsorption processes of surface O_2_ during UV light detection. Various improvement strategies have been proposed to overcome this drawback and keep the high sensitivity, including ZnO–organic semiconductor interfacing, defect engineering and doping, surface modification, heterojunction and the Schottky barrier. The general idea is to modify the adsorption state of O_2_ or replace the adsorbed O_2_ with another material to build a light-regulated surface or an interface barrier, as surveyed in the third section. The critical trade-off between sensitivity and response speed is also addressed. Finally, after a summary of these mechanisms and the improvement methods, this review is concluded with an outlook on the future development of ZnO nanomaterial UVPDs.

## 1. Introduction

As an important development in photoelectronic detection technology after infrared light detection, the detection of ultraviolet light has been intensely studied due to its potential applications in space astronomy, optical communication, missile detection, automatic navigation, high-voltage corona monitoring, flame detection and so on [1,2,3,4,5,6]. Furthermore, due to its wide applications in various industries—smartphone, automobile and robotics, for example—the market size of UVPDs is expected to increase by USD 7.57 billion in the next five years [7]. Various application scenarios have put forward different requirements for the performance of UVPDs; high sensitivity and the capability of weak light detection are required for space astronomical detection, optical communication and missile warning, while a quick response is also essential to automatic navigation and flame detection [8]. At present, various materials have been used: (1) Si [9,10,11], SiC [12,13] and GaN [14,15] with relatively mature commercial applications; (2) ZnO [16,17,18], NiO [19,20] and TiO_2_ [21,22] as relatively new materials; and (3) organic semiconductors [23,24,25] and perovskite [26,27,28], for example. These materials are applied in various devices: photoconductors [14,26], diodes (Schottky junctions [12,21], PN junctions [29], P-I-N [30], heterojunctions [9,20,23], avalanche diodes [31], drift diodes [32]), transistors and arrays prepared using diodes or transistors. However, high sensitivity and a quick response are generally limited in the above-mentioned devices, and the current research on UVPDs generally focuses on the selection of new materials and the design of new device structures to improve the sensitivity of sensors (such as single-photon detection) while ensuring the response speeds and expanding their application to flexible devices.

The sensitivity of UVPDs depend on the internal quantum efficiency of the material itself (the number of electron–hole pairs generated for each absorbed photon), the carrier lifetime, the light-regulated barrier height introduced by the device structure, the light-regulated potential (transistor), etc. The sensitivity can be characterized by the current on/off ratio, responsivity and detectivity. In order to improve the sensitivity of the sensor to UV light, it is usually necessary to introduce a light-regulated barrier or a built-in electric field (PN junction, heterojunction, etc.), but these structures will also introduce barrier capacitance and diffusion capacitance, which increases the transit time of carriers and reduces their response speed to UV light. At the same time, in order to increase the incident light irradiation area and reduce the dark current, the space between electrodes is usually increased, which will also lead to a longer carrier transport time and reduce the response speed. The response speed of the sensor to UV light is determined by factors such as carrier lifetime, carrier transport time, adsorption and desorption time (if gas adsorption is involved), etc. Therefore, reasonable material selection and device structure design are required to effectively regulate carrier transport and improve the sensitivity and response speed of a device to ultraviolet light.

To fulfill the above-mentioned detection criteria, ZnO nanomaterials are preferably used in UVPDs due to their unique electrical and optical properties [33]. As a natural n-type semiconductor with a wide direct bandgap (~3.37 eV), ZnO possesses a large exciton-binding energy of 60 meV at room temperature, which is beneficial for UV light detection [34]. Due to its rich defect characteristics, the band structure, defect level position and surface adsorption nature can be modified using various methods and are all crucial to UVPDs’ detection capabilities. Especially due to the adsorption of O_2_, ZnO nanomaterials usually exhibit a light-regulated surface barrier, which provides the possibility of ultrahigh UV light detection sensitivity. Other advantages of using ZnO materials in UVPDs can be further exemplified: the non-centrosymmetric structure enables Wurtzite ZnO with piezoelectric and pyroelectric properties, which can be utilized to improve the performance of UVPDs [35,36]. Due to its biocompatibility, ZnO is also a natural candidate in biomedical applications. Moreover, ZnO nanomaterials with different dimensions can be fabricated using various low-cost methods: chemical vapor deposition [17], the solution method [37], the sol-gel method [38] and magnetic sputtering, for example [39]. The available ZnO nanostructures include quantum dots [37,40], nanoparticles [41], nanowires [36,42], nanoflowers [43], nanosheets [44,45] and nanofilms [38], for instance. These nanostructures provide a large number of choices for promising nanoscale UVPDs applications, and the variety of low-cost fabrication methods is crucial to the further commercialization of ZnO UVPDs.

As a widely studied field, the complexity of ZnO UVPDs is growing with the rapid development of various device structures and detection strategies. To try to give a clear thread of the development, the underlying detection mechanisms of ZnO UVPDs with different device structures are firstly summarized as three basic ways in the second part: (2.1) the photoconductivity effect, (2.2) the threshold dimension effect and (2.3) the light-regulated interface barrier. A trade-off between sensitivity and response speed is observed in each of these mechanisms due to the adsorbed surface O_2_. To overcome this drawback, a natural idea is modifying the adsorption state of the O_2_ or replacing the surface O_2_ with other materials to build the surface/interface barrier. Various methods are proposed and highlighted in the third part, which includes (3.1) ZnO–organic semiconductor interfaces, (3.2) defect engineering and doping, (3.3) surface modification and (3.4) heterojunction and the Schottky barrier. With the detection mechanisms and their possible improvement expounded, this article is concluded with a summation of the possible future development trends of ZnO UVPDs.

## 2. Basic Detection Mechanisms

### 2.1. Photoconductivity Effect

**Figure 1:**
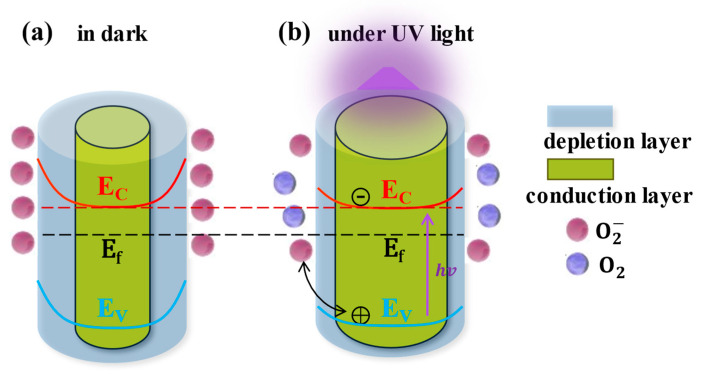
Photoconductive effect of ZnO. Reproduced from [16] with permission from the Royal Society of Chemistry, copyright 2019.

ZnO has a prominent advantage in improving sensitivity and realizing flexible devices: due to the large number of oxygen vacancies introduced during the growth of ZnO, there are a large number of defect states on its surface, which constitute adsorption sites of O_2_. Due to the existence of the defect states and the chemical adsorption of oxygen, the electron concentration on the ZnO surface will decrease, the surface energy band will bend upward and a depletion layer and a built-in electric field pointing from the inside to the surface are generated on the surface, as shown in Figure 1a. Correspondingly, the dark current of the ZnO would decrease due to this carrier depletion by surface adsorption. Under UV light illumination, the photogenerated electron–hole pairs inside the ZnO would be separated due to the surface band bend and built-in electric field; the photogenerated holes would drift to the surface and recombine with the negatively charged surface $O_{2}^{-}$ (Figure 1b). The recombination rate of the photogenerated electron–hole pairs would be correspondingly decreased, and the lifetime of the photogenerated electrons would be prolonged. The thickness of the surface depletion layer would also be reduced, and the electron concentration inside the ZnO would simultaneously increase. The increases in both the lifetime and concentration of the electrons would thereby lead to a large photocurrent, and this photoconductivity effect would be stronger than that of semiconductor materials without adsorption. However, the thickness of the depletion layer in ZnO nanomaterials is usually around 100 nm [16], and a conducting core would be left unaffected if a ZnO nanomaterial with a larger dimension were used. The response of UVPDs to the pure photoconductivity effect would be limited (usually ~100) due to the thickness ratio of the depletion layer to the conducting core.

### 2.2. Threshold Dimension Effect

**Figure 2:**
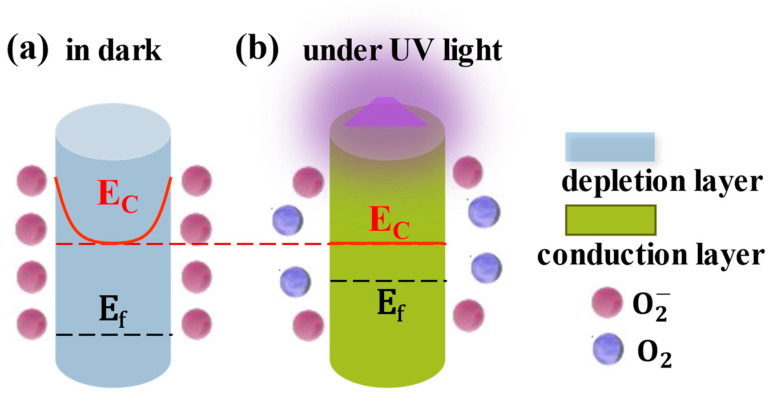
The threshold dimension effect of ZnO. Reproduced from [16]. with permission from the Royal Society of Chemistry, copyright 2019.

ZnO nanomaterials are generally used in UVPDs. Their small dimensions result in a large surface-to-volume ratio, which is crucial to the performance of ZnO UVPDs. Taking a ZnO nanoparticle, for example, with an assumed constant surface adsorption density of O_2_ ($n_{ad}$), the reduction in the dimensions (r, radius of nanoparticle) will lead to a depletion of ZnO electrons ($N_{dep}$) in proportion to the product of the surface adsorption density and surface area: $N_{dep}\sim n_{ad}r^{2}$. However, if the electron concentration of the ZnO ($n_{ZnO}$) is constant, the available electrons ($N_{total}$) would be proportional to the volume of the ZnO: $N_{total}\sim n_{ZnO}r^{3}.$ The ratio of depletion would be inversely proportional to the dimensions: $N_{dep}/N_{total}\sim 1/r$. This ratio also holds for nanowires or nanorods. In other words, with the reduction in the ZnO dimensions, the whole ZnO nanomaterial would be depleted eventually, and the Fermi level of the ZnO would decrease relative to the conduction band minimum. This phenomenon can also be explained from the energy band perspective (Figure 2): a constant surface adsorption density leads to a constant depletion width, but with a decrease in dimensions, the radius of the ZnO nanowires would be comparable to or smaller than the assumed depletion width. In this threshold situation, rather than further upward bending of the conduction and valence bands, the Fermi level would decrease, due to the increase in the depletion ratio, to the same height as that of the surface energy level due to surface adsorption: a similar phenomenon as Fermi level pinning.

**Figure 3:**
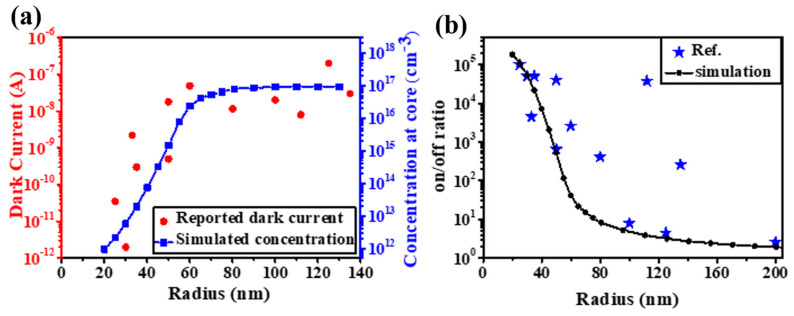
(**a**) Reported dark currents versus radii of ZnO nanowires and their relations with the concentrations at the core. (**b**) Reported on/off ratios versus the radii of ZnO nanowires. Reproduced from [16] with permission from the Royal Society of Chemistry, copyright 2019.

The distribution of an electric field and a carrier concentration with a constant surface adsorption density can be simulated by a space-charge model and the surface potential measured by Kelvin probe force microscopy (KPFM) [16]. According to this model, 0.3 eV surface band bending would result in a depletion layer with ~80 nm thickness. Correspondingly, ZnO nanomaterials with dimensions smaller than this threshold would be completely depleted by surface-adsorbed oxygen. In other words, with the decrease in the dimensions, the carriers inside the ZnO core besides the surface carrier would also be involved in the adsorption process, which would result in the enlargement of the distance between the Fermi level ($E_{f}$) and the core conduction band ($E_{C}^{c}$). An exponential decrease in the carrier concentration would be expected. The carrier concentration would reverse to its original level when the adsorbed oxygen is desorbed due to UV light illumination and UVPDs with a ~10^5^ on/off ratio is realized (threshold dimension effect) [16]. This effect can be verified by the dramatic decreases in the dark currents of different ZnO nanowire UVPDs reported in the literature. Although different growth and measurement conditions have been used, a sharp drop in the dark current related to complete depletion due to the threshold dimension effect is clearly observed when the radius is below the threshold dimension, as shown in Figure 3. The decrease in the dark current (Figure 3a) and the increase in the on/off ratio (Figure 3b) due to this effect are compared to the simulated results based on the space-charge model.

### 2.3. Light-Regulated Interface Barrier

**Figure 4:**
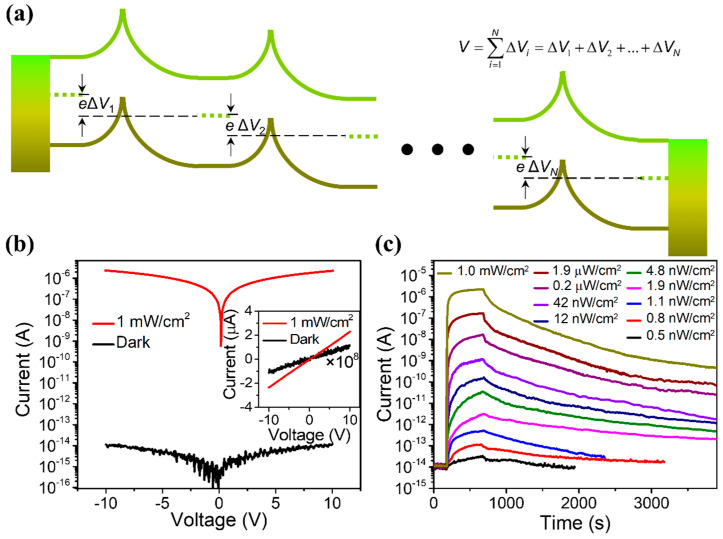
(**a**) This band diagram of the fiber-based ZnO nanowire (NW) network UV photodetector indicates that the applied voltage across the whole structure is separated by the high number of NW junctions, as the inset equation shows, and a small voltage across each interface is ensured. (**b**) Response to 1.0 mW cm^−2^ UV light. The inset indicates good linear behavior both in the dark and under UV illumination. (**c**) Response to UV light with different intensities from 0.5 nW cm^−2^ to 1.0 mW cm^−2^. Reproduced from [17] with permission from the American Chemical Society, copyright 2020.

In addition to the above-mentioned effects, the depletion layer at the ZnO–ZnO interface can also be used to construct a light-regulated interface barrier, which can further sharply reduce the dark current of the device and improve the sensitivity of the UVPDs. When irradiated with ultraviolet light, the barrier height will decrease and the photocurrent will increase exponentially (light-regulated barrier, Figure 4a). The current density is exponentially proportional to this interface barrier height, as explained by the back-to-back Schottky model. Consequently, the on/off ratio of the UVPDs would be exponentially proportional to the change in the interface barrier height due to the UV light illumination (${e∆\phi =e\phi }_{UV}-e\phi_{dark})$. According to this model, a 0.3 eV change in barrier height would lead to an on/off ratio with a ~10^5^ magnitude [17]. Furthermore, a large number of interface barriers can be constructed using the multi-interface structure, such as the ZnO nanowire network and ZnO nanofilm, for example, which can further increase the modulation effect of the light-regulated interface barrier. Combining the above three effects, the on/off ratio of the ultraviolet sensor prepared using the ZnO network structure can reach more than 10^8^, which is much higher than those of commercially used Si, GaN, organic semiconductors and perovskite materials, as demonstrated in Figure 4b,c.

However, this high sensitivity is realized with costs: due to the adsorption of the O_2_ on the ZnO surface and interface, the response speed of theUVPDs is dramatically limited by the slow desorption and re-adsorption processes during UV light illumination and recovers in the dark cycle, as shown in Figure 4c. To keep the high on/off ratio of the ZnO UVPDs and increase its response speed, a natural solution is to replace the surface-adsorbed O_2_ with other materials, which can keep the threshold size effect and the light-regulated interface barrier while reducing the desorption and re-adsorption on the surface. Different methods have been proposed, as discussed below.

## 3. Methods of Improvement

### 3.1. ZnO–Organic Semiconductor Interfaces

The combination of inorganic and organic semiconductors is a possible solution for the realization of UVPDs with high sensitivity and fast response speeds. Organic semiconductor molecules bonded by π bonds and van der Waals forces exhibit the highest occupied and lowest unoccupied molecular orbitals (HOMOs/LUMOs). The large energy gap difference makes a very low concentration of thermally excited electron–hole pairs, which usually leads to a very low dark current of the organic semiconductor sensors. At the same time, since organic semiconductors usually absorb light more strongly than inorganic semiconductors, organic semiconductors with suitable band gaps have stronger detection capabilities for weak UV light [46]. In addition, by spin coating or printing on flexible substrates, flexible UV sensors can be fabricated and applied to smart sensing, wearables and flexible devices. However, since organic semiconductors realize carrier transport through transitions between molecular orbitals, their carrier mobilities (10^−6^~20 cm^2^ · V^−1^ s^−1^) are usually much smaller than those of inorganic semiconductors (60~1000 cm^2^ · V^−1^ s^−1^) [47]. Additionally, because of the large binding energy of the excitons (0.1~2.0 eV), photogenerated electron–hole pairs are difficult to separate, which is not conductive and cannot contribute to the sensors’ photocurrent. UVPDs using organic semiconductors would hence require suitable donor and acceptor interfaces to achieve the separation and transport of electron–hole pairs [1]. In terms of device structure, in order to increase the illumination area and reduce the dark current, large electrode spacing is generally required, but this will lead to a longer carrier transport distance and thus increase the sensor’s response time to UV light. Using a vertical layered structure can reduce the carrier transport distance to the thickness of the device, thereby reducing the carrier transport time. However, the layered structure and the penetration of the electrode into the organic semiconductor materials will lead to an increase in the dark current and, consequently, further reduction in the sensor’s on/off ratio.

**Figure 5:**
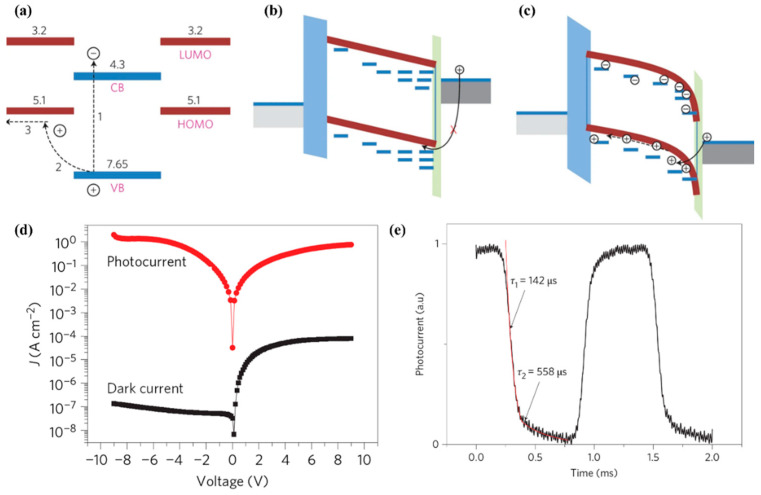
(**a**) Energy diagram of the nanoparticle with the surrounding polymer. CB, conduction band; VB, valence band; LUMO, lowest unoccupied molecular orbital; HOMO, highest occupied molecular orbital. (**b**,**c**) Energy diagram of the device in the dark (**b**) and under illumination (**c**). (**d**) Photocurrent and dark current density of the PVK:ZnO device. (**e**) Transient photocurrent of the P3HT:ZnO device. Adapted from [1]. Copyright 2012, Springer Nature. Available under the STM Permissions Guidelines, https://www.stm-assoc.org/intellectual-property/permissions/permissions-guidelines/ (accessed on 25 March 2025).

The combination of ZnO and organic semiconductors can effectively solve the above problems. ZnO can act as an extraction and transport layer for photogenerated electrons and reduce the device’s dark current [48]. For example, in organic semiconductor heterojunction UV sensors, after reduction of the concentration of ZnO surface defect states by chemical–thermal treatment, ZnO, as an electron transport layer, could improve the sensor response speed, and the response time to UV light was reduced to the order of 100 μs [49]. At the same time, the large number of interface states on the ZnO surface can effectively regulate the injection of photogenerated carriers into the transport channel, thereby increasing the photocurrent (an effect similar to that with the light-regulated interface barrier). For example, polyvinyl carbazole (PVK) and ZnO nanoparticles can be mixed to act as a photosensitive layer. Photogenerated electrons are accumulated inside the ZnO due to its nanometer size and a large number of surface states. At the same time, due to the enrichment of the ZnO on the cathode side during the mixing of the ZnO and PVK, the accumulation of the photogenerated electrons inside the ZnO particles near the cathode side will result in an electrical potential drop on the cathode side as well as a decrease in the hole injection barrier from the cathode. Thereby, the ZnO nanoparticles act as a light-regulated hole-injection “valve” in the UVPDs (Figure 5a–c). As a result, the hole injection efficiency will increase, and an on/off ratio in the magnitude of 10^7^ will be realized. Due to the elimination of the O_2_ desorption and re-adsorption processes in UVPDs, the response to UV light will also be accelerated, around the order of 500 μs (Figure 5d,e) [1]. A similar method is also used in the PEDOT:PSS-C_60_ UVPDs, where the surface defect states of ZnO are utilized as the regulating sites of charge injection. A UV light detection limit of as low as 10 fW/cm^2^ has consequently been accomplished [50].

### 3.2. Defect Engineering and Doping

**Figure 6:**
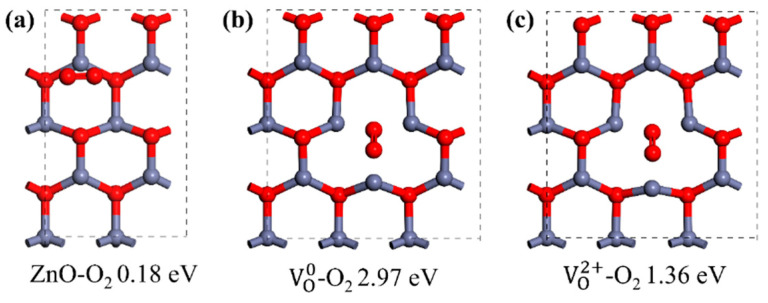
Adsorption geometry configuration and adsorption energy of O_2_ on (**a**) ZnO surface, (**b**) ZnO surface with $V_{O}^{0}$ and (**c**) ZnO surface with $V_{O}^{2+}$. Adapted from reference [18] with permission from Elsevier, copyright 2020.

Usually, n-type conductivity is exhibited by as-grown ZnO materials and caused by intrinsic defects and unintentional doping. Intrinsic and extrinsic defects are crucial to the UV response of ZnO. Taking oxygen vacancy (V_O_), for example: due to its relative low formation energy during growth, a large quantity of V_O_ is usually expected in ZnO and acts as a donor-type defect [51,52]. At room temperature, electrons would transfer from the V_O_ donor level to the conduction band, resulting in n-type conductivity [53]. For UVPDs, a high concentration of V_O_ is generally related to a large dark current if its presence on the ZnO surface is not taken into consideration. However, due to the large surface-to-volume ratio of ZnO nanomaterials, the adsorption and desorption processes of atmosphere O_2_ are also influenced by V_O_. Compared to a bare ZnO surface, the presence of V_O_ would lead to a larger adsorption energy and a higher portion of electron transfer between adsorbed O_2_ and ZnO, which means a larger adsorption surface density and, consequently, a higher surface barrier height resulting from the adsorbed O_2_ [54]. The photoconductivity effect, threshold dimension effect and light-regulated interface barrier would all be more dramatic. As a result, an increase in the V_O_ concentration is generally related to an increase in the on/off ratio and a decrease in the response speed of ZnO UVPDs. Furthermore, the state of V_O_ also plays a crucial role in ZnO UVPDs; as a negative U center, the charge state of V_O_ can vary from neutral ($V_{O}^{x}$) to doubly ionized ($V_{O}^{++}$), depending on the growth condition and the Fermi level position [18]. Oxygen vacancies with different charge states show different adsorption energy and geometric configurations, which control the adsorption and desorption processes of atmosphere O_2_, as shown in Figure 6. What is more: the charge state also determines the defect level position of the V_O_ inside the ZnO band gap, which, in turn, influences the generation and recombination rates of electron-and-hole pairs.

The above V_O_ example shows that the defect concentration and defect state in ZnO have decisive influence on its UV detection performance. Therefore, various methods are proposed to regulate its defects. For intrinsic defects, the concentration and state of the defects can be controlled by changing the growth conditions or post-annealing in different atmospheres. For example, the post-annealing of ZnO nanofilm in the air and at 400 °C can promote the transformation of $V_{O}^{x}$ in ZnO to $V_{O}^{++}$, thereby increasing the adsorbed oxygen density on the surface of the ZnO and increasing the on/off ratio of UVPDs [18]. Using the hydrothermal method or annealing within H_2_ gas, H atoms will first combine with V_O_ and zinc vacancies (V_Zn_) in ZnO to passivate them, thereby inhibiting the activity of these intrinsic defects and reducing the recombination probability of carriers by defects during electron transitions [55]. This phenomenon is generally related to a decrease in the green luminescence intensity of the photoluminescence spectrum of the ZnO [56]. However, further hydrothermal treatment would result in the breaking of the Zn-O bonds and the removal of O atoms from the ZnO due to the generation of H_2_O [57]. Additional V_O_ and hydrogen interstitials (H_i_) would consequently be generated. Both defects would lead to an increase in carrier concentration, thereby increasing the photocurrent of the UVPDs.

**Figure 7:**
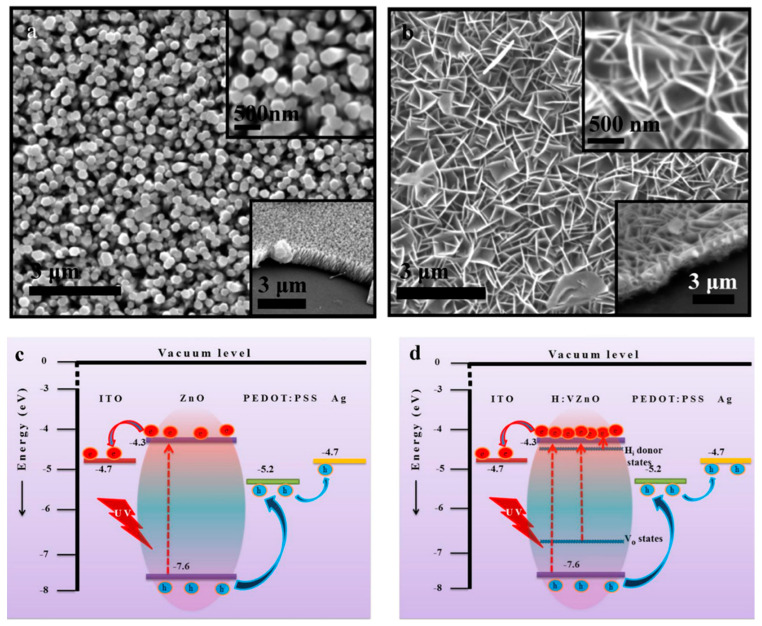
SEM images of (**a**) pristine ZnO NRs and (**b**) VZnO NFs. Energy band diagrams of self-powered (**c**) pristine ZnO NRs and (**d**) H:VZnO NF UV photodetectors. Adapted from reference [66] with permission from the American Chemical Society, copyright 2016.

Besides the additional donor or acceptor defects, doping with different elements can also result in the regulation of intrinsic defects at the same time. Doping with elements such as Ga [58], Al [59] and Co [60] will lead to an increase in the V_Zn_ concentration, while the doping of elements such as La [61], Mn [62] and Cr [63], with larger ion radii than that of a Zn ion, would lead to increasing internal stress due to lattice perturbation and reconstruction. This internal stress would be released by the formation of V_O_ and Zn_i_ during growth. Due to the larger coordination number than that of Zn, doped Mn and Fe atoms will capture O atoms from Zn atoms in the lattice, resulting in the generation of V_O_ around the Zn atoms [64,65]. In addition, the introduction of donor-impurity atoms will also increase the electron concentration in ZnO and thus increase the photocurrent. Furthermore, doping can also lead to morphology changes during growth. Due to the strong attachment of ${V(OH)}_{4}^{-}$ with a Zn^2+^-terminated polar surface during hydrothermal growth, lateral growth rather than growth along the c-axis of ZnO is promoted, and the morphology of ZnO will change from nanowire arrays (NRs) to nanoflake arrays (NFs), which is beneficial to light-harvesting efficiency during UV light detection due to the smaller light scattering from the planar structure and multiple reflections inside its cavity (Figure 7a,b) [43,66]. Compared to pristine ZnO, a large amount of shallow H_i_ donor levels and deep V_O_ donor levels are generated due to doping, and hydrogenation and a higher electron concentration in the conduction band are generated during UV light illumination (Figure 7c,d).

The response speed of ZnO UVPDs is limited by the recombination and release of photogenerated carriers, which are usually determined by a fast process at the deep energy level and a slow process on the surface energy level. The introduction of intrinsic defects and impurity atoms will lead to an increased concentration of the deep levels in the bandgap, and the recombination rate of the photogenerated carriers will be consequently increased. In other words, the replacement of a surface defect level with a deep level is beneficial to the response speed. In the above example, a small response time, around 20 microseconds, has also been observed, which can be ascribed to intrinsic defect generation by V-doping and hydrogenation [66]. However, the concentration of the surface defects will simultaneously increase, which will result in a larger amount of adsorbed oxygen and a slowed-down response to UV light. Furthermore, the doping process during growth will also change the surface morphology of the ZnO nanomaterials, then affecting the adsorption and desorption rate of the oxygen. Therefore, these factors should all be taken into consideration to account the impact of doping on the response speed of UVPDs, and the trade-off between sensitivity and response speed should still be balanced by defect engineering and doping.

### 3.3. Surface Modification

The adsorption and desorption of atmosphere O_2_ on the ZnO surface are beneficial to the sensitivity of ZnO UVPDs but detrimental to their response speed due to the slow nature of these processes. In other words, it is contradictory to simultaneously obtain an ultra-high on/off ratio and a fast response speed by regulating the surface oxygen adsorption process. Therefore, it is a natural idea to replace the role of oxygen adsorption by modifying the metal atoms and organic molecules on the surface of the ZnO; a Schottky contact will be formed between the metals and the ZnO if each metal has a work function greater than that of the ZnO. Similar to the depletion layer resulted from the adsorbed O_2_, this Schottky barrier will also lead to upward band bending and an inner electric field, which will reduce the carrier concentration in the dark. Under UV light illumination, the photogenerated holes in the ZnO will move to the surface due to the built-in electric field of the bent surface energy band, thereby reducing the probability of the recombination of the photogenerated electrons and holes. The lifetime and mobility of the photogenerated electrons will be increased and the photocurrent will be enhanced [67]. Therefore, the Schottky barrier near the ZnO surface can also result in the threshold dimension effect while avoiding the slow adsorption and desorption processes.

**Figure 8:**
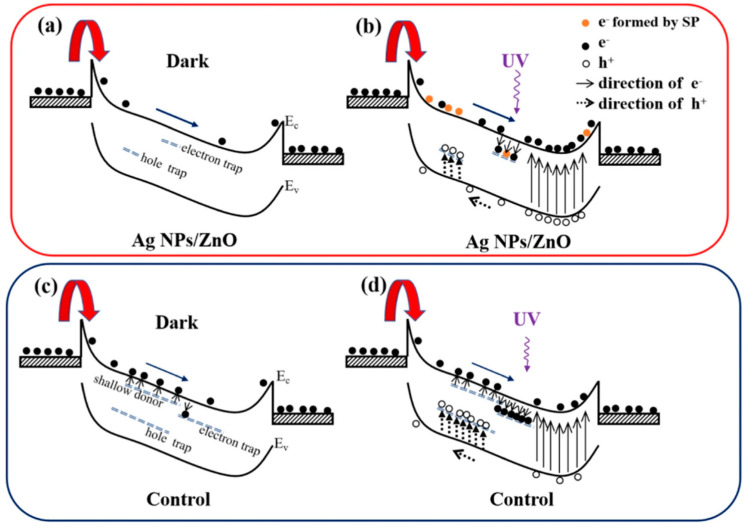
(**a**,**b**) Schematic diagram for carrier transportation and generation of ZnO film with Ag nanoparticles (NPs) in the dark and in 365 nm illumination, respectively. (**c**,**d**) Schematic diagrams of carrier transportation and generation of ZnO film without Ag NPs in the dark and in 365 nm illumination, respectively. Reproduced from [69]. Springer Nature, copyright 2020. Available under the Creative Commons Attribution 4.0 Generic License, https://creativecommons.org/licenses/by/4.0/ (accessed on 25 March 2025).

However, the excessive adsorption of metal atoms may also cause short-circuiting on the ZnO surface, thereby reducing the effect of the light regulation barrier, so it is necessary to control the surface density of the metal atoms adsorbed on the surface. Generally, it can be adjusted by changing the parameters, such as the metal solution concentration [68], the electric field frequency [69] and the time length of the dielectrophoretic process [67]. The modification of the metal nanoparticles will also introduce surface plasmon under UV irradiation, thereby improving the absorption and response of the UVPDs to UV light. At the same time, the defect state of the ZnO surface may also be regulated by the metal particles adsorbed on the surface; due to the passivation of the ZnO surface by metal particles such as Ag atoms and the substitution of Ag atoms for surface V_Zn_, the density of the oxygen and -OH adsorbed on the ZnO surface will decrease, thereby improving the response speed of the UVPDs, as illustrated in Figure 8 [69]. Similarly, the surface modification of ZnO with carbon quantum dots can reduce the density of the surface-adsorbed oxygen and thus improve the response speed [38]. Using surface modification, a response time of around 10 s and an on/off ratio of around 10^5^ can be achieved [38,67,69,70,71,72].

### 3.4. Heterojunction and the Schottky Barrier

**Figure 9:**
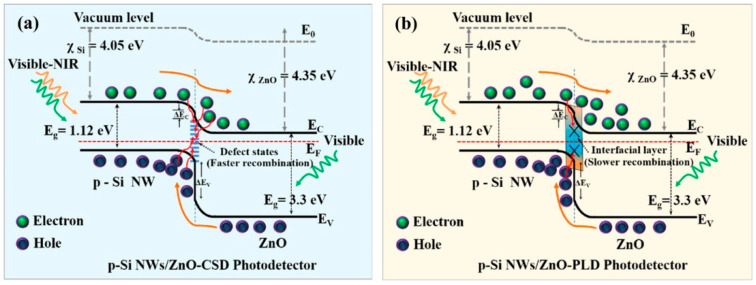
Schematic of the energy band diagram for photoexcited charge transport by taking into account the role of the interface defect states within the p-n junction for (**a**) CSD- and (**b**) PLD-grown heterostructure arrays of p-Si NWs/ZnO photodetectors. The transportation and recombination of photogenerated carriers are represented by the brown and red arrows respectively. Reprinted with permission from [74]. Copyright 2023, American Chemical Society.

Due to the presence of adsorbed O_2_ on the ZnO surface, the barrier of the ZnO–ZnO homojunction is limited by the slow process of desorption and re-adsorption, which would dramatically constrain the response speed of UVPDs. The heterojunction formed by combining ZnO with other semiconductors or a Schottky barrier with metal can replace the ZnO–ZnO homojunction to build a light-regulation barrier [36,73]. Due to the built-in electric field of the heterojunction or Schottky barrier, the photogenerated carriers are separated, thereby reducing the recombination of the photogenerated carriers and improving the sensitivity of the UVPDs, as illustrated by the heterojunction of the p-Si/n-ZnO UVPDs in Figure 9 [74]. This separation is also influenced by the surface defect states on the ZnO surface, which act as fast recombination centers of photogenerated carriers and reduce the photocurrent under UV light illumination. Compared to chemical solution deposition (CSD), a reduced density of surface defect states and, consequently, a higher sensitivity of ZnO UVPDs are obtained by pulsed laser deposition (PLD). At the same time, the formation of a heterojunction can reduce the contact between the ZnO surface and the oxygen in the air and reduce the adsorption density, thereby increasing the response speed. In addition, the response of other semiconductor materials to UV light can also be used to increase the photocurrent. For example, Cs_3_Cu_2_I_5_ has a strong absorption of deep UV light, with wavelengths near 260 nm. Combined with the response of ZnO to the UV light, the UV detection band of the UVPDs can be improved [37]. With a surface/interface barrier resulting from a heterojunction or Schottky barrier rather than surface-adsorbed O_2_, a response time of around 20 ms can be achieved [36]. What is more: this surface/interface barrier from heterojunction also offers the possibility of improvement by the piezoelectric or pyroelectric field of ZnO [35,36]. Another advantage of heterojunction is that by choosing the heterojunction type, the spontaneous formation of 2D electron gas near the junction, which exhibits a high carrier mobility and carrier concentration, can be achieved. The response speed and sensitivity of the UVPDs can, consequently, be improved. For example, the ZnO/ZnMgO heterostructure UVPDs has an on/off ratio of around 10^7^ [75].

## 4. Summary and Perspective

To sum up, the detection mechanisms of UVPDs based on ZnO nanomaterials can be generally classified as three basic types: (1) the photoconductivity effect—the photoconductivity of ZnO is enhanced by the UV-modulated surface depletion layer due to adsorbed O_2_; (2) the threshold dimension effect—the complete depletion of ZnO with dimensions under a threshold, resulting in a dramatic UV light modulation of its carrier concentration; and (3) the light-regulated interface barrier—when surface depletion and band bending compose a UV light-regulated interface barrier that exhibits an exponential modulation of the current density. ZnO UVPDs with on/off ratios exceeding 10^8^ can be achieved by a combination of the above effects. However, the response speed would be generally restricted by the slow desorption and re-adsorption processes during UV light illumination and recovered in the dark cycle. Different methods have been proposed to overcome this restriction: (1) A combination of ZnO and an organic semiconductor can utilize the advantages of the high electron mobility of the ZnO and the high sensitivity of the organic semiconductor simultaneously. Photogenerated electron–hole pairs can be separated by the interface. (2) By defect engineering and doping, neutral oxygen vacancy can be transformed into doubly ionized states with smaller adsorption energy values to facilitate the adsorption and desorption processes. Doping can regulate intrinsic defects and result in a high carrier concentration and a suitable morphology of ZnO nanomaterials with enhanced light-harvesting efficiency. (3) Surface modification with adsorbed metal particles can replace the role of adsorbed O_2_ during UV light detection with reduced surface vacancies and a high response speed. (4) The light-regulated barrier can also be constructed by heterojunction or a Schottky barrier to facilitate the separation of photogenerated carriers.

The trade-off between sensitivity and response speed can be relatively overcome by these methods, and a response time of around 1 ms or 0.1 ms is possible. However, several issues still need to be addressed in the further development of ZnO UVPDs: (1) The response times of ZnO UVPDs are still much larger than those of Si photodiodes, which are typically around 100 ns or less. To achieve this goal, the native defects in ZnO should be further reduced due to the character of their trapping center during the generation and recombination of photocarriers. Moreover, new interface defect states will be formed if a ZnO–organic semiconductor interface, surface modification or heterojunction is used. The reduction of these interface states should also be investigated. (2) To commercialize the ZnO UVPDs, long-time stability should be studied in various device structures. Especially due to the high energy and aging effect of UV light, possible material degeneration should be avoided. (3) As a highly investigated direction, the performance of self-powered ZnO UVPDs is still limited, especially their sensitivity compared with conventional ZnO UVPDs. To enhance this sensitivity, the separation efficiency of photogenerated carriers by built-in electric fields should be further improved.

## Data Availability

No new data were created or analyzed in this study. Data sharing is not applicable to this article.
